# Defining the progeria phenome

**DOI:** 10.18632/aging.205537

**Published:** 2024-02-09

**Authors:** Cecilie Worm, Maya Elena Ramirez Schambye, Garik V. Mkrtchyan, Alexander Veviorskiy, Anastasia Shneyderman, Ivan V. Ozerov, Alex Zhavoronkov, Daniela Bakula, Morten Scheibye-Knudsen

**Affiliations:** 1 Center for Healthy Aging, Department of Cellular and Molecular Medicine, University of Copenhagen, Denmark; 2 Insilico Medicine AI Limited, Level 6, Unit 08, Block A, IRENA HQ Building, Masdar City, Abu Dhabi, UAE; 3 Insilico Medicine Hong Kong Limited, Science Park West Avenue, Hong Kong, China

**Keywords:** aging, progeria, premature aging, phenome, clinical phenotype

## Abstract

Progeroid disorders are a heterogenous group of rare and complex hereditary syndromes presenting with pleiotropic phenotypes associated with normal aging. Due to the large variation in clinical presentation the diseases pose a diagnostic challenge for clinicians which consequently restricts medical research. To accommodate the challenge, we compiled a list of known progeroid syndromes and calculated the mean prevalence of their associated phenotypes, defining what we term the ‘progeria phenome’. The data were used to train a support vector machine that is available at https://www.mitodb.com and able to classify progerias based on phenotypes. Furthermore, this allowed us to investigate the correlation of progeroid syndromes and syndromes with various pathogenesis using hierarchical clustering algorithms and disease networks. We detected that ataxia-telangiectasia like disorder 2, spastic paraplegia 49 and Meier-Gorlin syndrome display strong association to progeroid syndromes, thereby implying that the syndromes are previously unrecognized progerias. In conclusion, our study has provided tools to evaluate the likelihood of a syndrome or patient being progeroid. This is a considerable step forward in our understanding of what constitutes a premature aging disorder and how to diagnose them.

## INTRODUCTION

The first case of a disease displaying signs of premature aging was reported by dermatologist Dr. Moritz Kaposi who described the syndrome Xeroderma Pigmentosum in 1874 [1]. He presented case reports of several young individuals who suffered from severe skin abnormalities commonly associated with aging of the skin. In 1886 Dr. Jonathan Hutchinson described a disease displaying signs of premature aging in a 3.5-year-old boy who presented with ‘a very peculiar and old-mannish look’ [2]. Hutchinson further provided a detailed description of the boy’s phenotypic features including a large head, open anterior fontanel, thin scalp and skin, lack of subcutaneous fat, alopecia, prominent veins, and muscle atrophy. Due to the striking overlap with normal aging, Dr. Hastings Gilford introduced the term progeria (Greek for prematurely old) and later the syndrome was referred to as Hutchinson-Gilford Progeria syndrome (ref: Gilford, Hastings. “Progeria: a form of senilism.” Practitioner 73 (1904): 188-217). Several progeroid syndromes have since been identified, and the information coming from understanding the pathogenesis of the syndromes has proven to be of great value in the research of normal aging. For instance, the identification that loss of DNA repair in multiple disease leads to premature aging has contributed to adding “genomic instability” as a hallmark of aging [3]. Strikingly, almost all progerias have been associated with genome instability while loss of other hallmarks in general have not been considered leading to a progeria phenotype [4]. However, what constitutes a progeria is not well defined and there are few tools to identify a progeroid patient. Furthermore, progeroid diseases are generally difficult to diagnose due to the large variations in clinical presentation [5]. Notably, whole exome sequencing only leads to diagnoses in 30-50% of patients [6] suggesting additional diagnostic strategies are needed.

In this study, we have utilized phenome explorations to define the phenotypes associated with progerias and to develop tools to diagnose patients and identify new progeroid syndromes. We compiled a list of known progerias and manually curated literature describing phenotypes associated with each disease. We then performed agglomerative hierarchical clustering, network investigations and principal component analysis on this data to identify correlations between disorders of different etiology. This allowed us to define the average phenotypes (the progeria phenome) of the progeroid patients and compare it with diseases that have been associated with premature aging (mitochondrial, autophagy and DNA repair disorders). In sum, we have defined what a premature aging disease is and developed tools to allow diagnostics of patients and disease population.

## RESULTS

### Identification of progeroid syndromes

**Figure 1:**
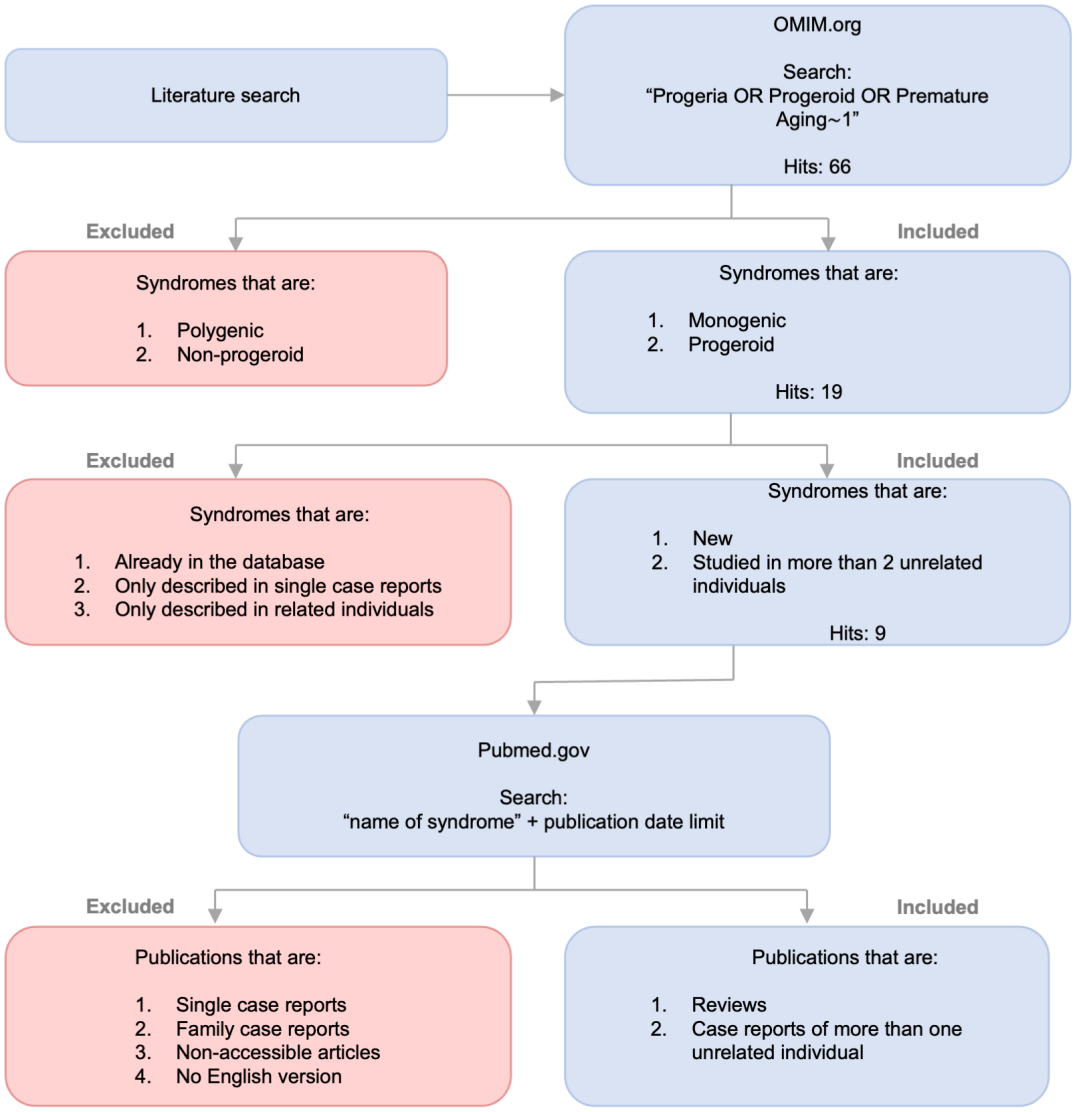
**Identification of progeroid syndromes for database.** Flow diagram illustrating the process of identifying new progeroid syndromes for mitodb.com including search, inclusion and exclusion criteria.

The syndrome database on https://www.mitodb.com was used in this study as a source of data concerning known progeroid syndromes and other groups of syndromes. To first identify premature aging diseases previously unrecognized by the mitodb database, putative progeroid diseases in the Online Mendelian Inheritance in Man (OMIM) were identified using the search strategy ‘Progeroid’, ‘Progeria’ and ‘Premature aging’. Hits were sorted based on a predefined inclusion and exclusion criteria allowing the identification of 32 premature aging syndromes (Figure 1 and Table 1). For each disease, published papers on PubMed were identified and prevalences of phenotypes in each disease were added to the database (see Supplementary Figures 1, 2 and Methods “Mean Prevalence of Phenotypes”). This quantified description of each disease allowed to compare and cluster diseases and phenotypes from all known progerias similar to what we have previously done with mitochondrial disorders [7].

**Table 1:** The progeroid syndromes.

**Syndrome**	**Gene**
Branchiooculofacial Syndrome	TFAP2A
Acromicric Dysplasia	FBN1
Ataxia-Telangiectasia	ATM
Bloom Syndrome	BLM
Cockayne Syndrome	ERCC6
Dyskeratosis Congenita	TERC
Fanconi Anemia	FANCA
Fontaine Syndrome	SLC25A24
GAPO Syndrome	ANTXR1
Geroderma Osteodysplasticum	GORAB
Hutchison-Gilford Progeria Syndrome	LMNA
Keppen-Lubinsky Syndrome	KCNJ6
Mandibular hypoplasia, Deafness, Progeroid features, and Lipodystrophy Syndrome	POLD1
Marbach-Rustad Progeroid Syndrome	LEMD2
Nestor-Guillermo Progeria Syndrome	BANF1
Nijmegen breakage Syndrome	NBS1
Penttinen Syndrome	PDGFRB
Rahman Syndrome	HIST1H1E
Rothmund-Thomson Syndrome	RECQL4
Ruijs-Aalfs Syndrome	SPRTN
Saul-Wilson Syndrome	COG4
Seckel Syndrome	ATR
Seckel Syndrome 2	RBBP8
Short stature, Hyperextensibility, Hernia, Ocular depression, Rieger anomaly, and Teething delay Syndrome	PIK3R1
Werner Syndrome	WRN
Wiedemann-Rautenstrauch Syndrome	POLR3A
Xeroderma Pigmentosum group A	XPA
Xeroderma Pigmentosum group B	XPB
Xeroderma Pigmentosum group C	XPC
Xeroderma Pigmentosum group E	XPE
Xeroderma Pigmentosum group F	XPF
Xeroderma Pigmentosum group G	XPG
Xeroderma Pigmentosum group V	XPV

Table listing the progeroid syndromes by name and respective affected gene.

### Hierarchical clustering identifies five phenotypical groups of progerias

**Figure 2:**
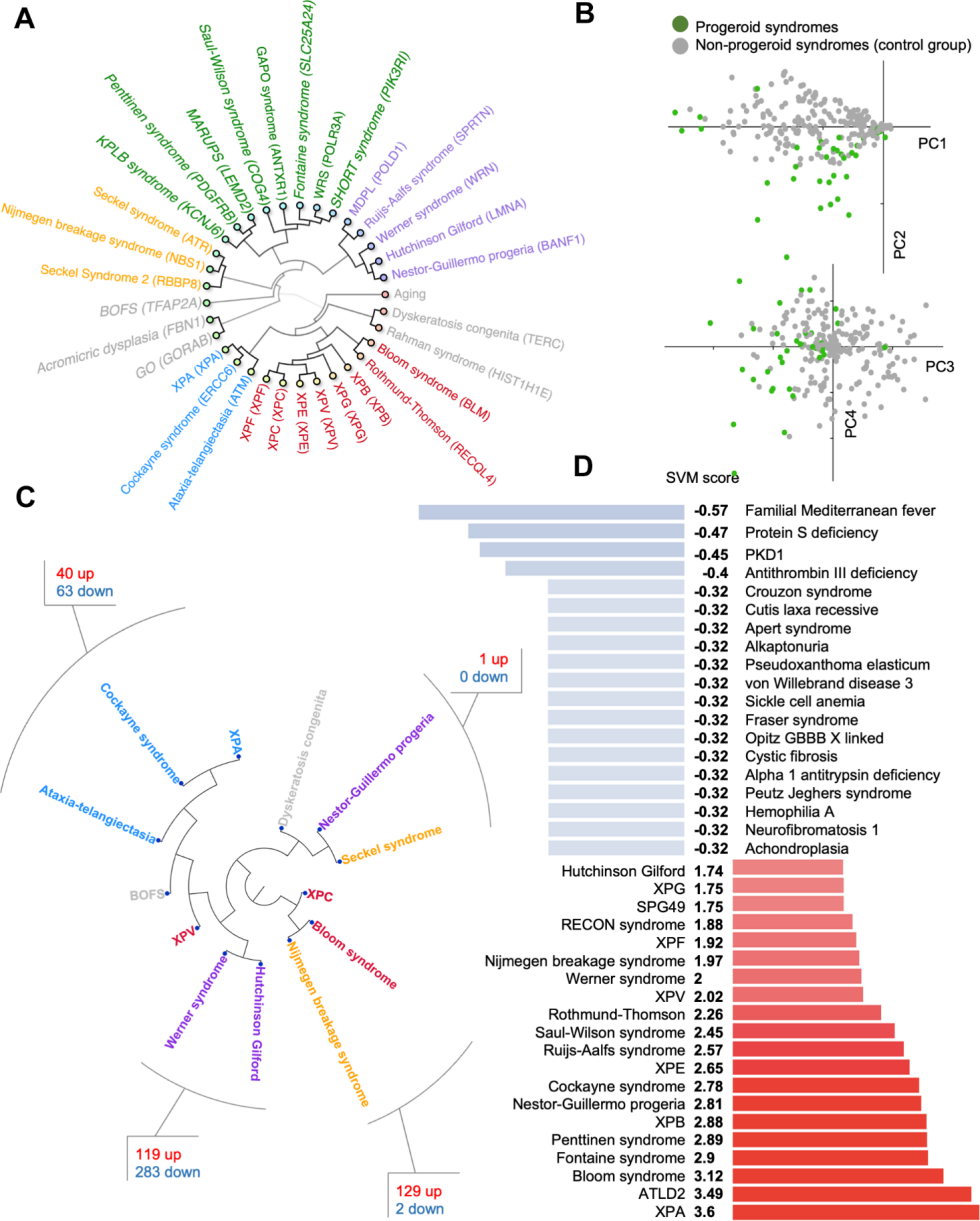
**Progeroid syndrome overview.** (**A**) Agglomerative hierarchical cluster based on phenotype prevalences using uncentered similarity and average linkage. (dark green are new to the database, other colors represent different clusters). Each syndrome group is color coded in the inner circle. (**B**) Principal component analysis of diseases based on the prevalence of phenotypes. (**C**) Hierarchical clustering of publicly available dataset for some premature aging diseases and the shared pathways between closely associated diseases. (**D**) Support vector machine scores for premature aging diseases (available at https://www.mitodb.com).

To understand the overlap and differences in clinical phenotypes between these rare diseases, principal component analysis, hierarchical clustering and networking tools were used on all 32 progeroid syndromes in the database. We also included normal human aging that consists of the human phenome as previously defined by us [8]. The hierarchical clustering (Figure 2A) showed progeroid syndromes clustered in groups indicating phenotypic diversity: one group contained syndromes such as Cockayne syndrome, Xeroderma Pigmentosum A (XPA) and Ataxia-Telangiectasia sharing phenotypes like cerebellar atrophy and short stature, a second group contained syndromes such as Werner syndrome, Rujis-Aalfs syndrome and Hutchinson Gilford sharing phenotypes like micrognathia and short stature, a third group contained Seckel syndrome and Nijmegen breakage sharing phenotypes like microcephaly, developmental delay and prominent nose, a fourth group contained Bloom syndrome and Xeroderma Pigmentosum (B, C, F, G, V and E) sharing phenotypes like cancer and sun sensitivity and a fifth group contained syndromes such as KPLB syndrome, GAPO syndrome and SHORT syndrome sharing phenotypes like micrognathia and skin wrinkles. Brachiooculofacial syndrome (BOFS), Geroderma osteodysplasticum (GO) and Acromicric dysplasia deviated from the other syndromes indicating a substantial difference in phenotypic traits and perhaps a weaker progeria phenotype.

To identify potential outliers in our dataset we used Principal Component Analysis (PCA) of the quantified traits (Figure 2B). Surprisingly, the PCA did not result in a strong separation between diseases, indicating that there are no clear phenotypic differences in the dataset. While the analysis indicated a considerable variation in the clinical presentation, it did not provide evidence to exclude any syndrome from our dataset.

### Transcriptomics reveals altered pathways in premature aging diseases

To understand if the clinical phenotypes could be reflected in molecular changes, we explored gene expression data from premature aging diseases. We were able to identify published gene expression data from 13 progeroid syndromes allowing us to investigate the similarity between diseases (Figure 2C). Interestingly, we observed that Cockayne syndrome, Xeroderma Pigmentosum group A (XPA), and Ataxia-Telangiectasia, three diseases associated with premature neurological aging, formed a close cluster. Pathway analysis revealed that DNA damage and multiple inflammatory processes were common between these diseases (Supplementary Table 1). For Werner syndrome and Hutchinson Gilford Progeria Syndrome pathway analysis showed perturbation of growth signals and the DNA damage response (Supplementary Table 2). Further, Bloom syndrome was found to cluster with Nijmegen breakage syndrome supporting their biochemical role in double stranded DNA break repair and shared pathways were growth signaling, DNA damage response pathways, transcription and inflammation (Supplementary Table 3). Lastly, Seckel syndrome clustered together with Nestor-Guillermo progeria and dyskeratosis congenita perhaps suggesting issue with DNA replication and cell division leads to similar transcriptional outcomes. However, almost no pathways were found to overlap between Nestor-Guillermo progeria and Seckel syndrome (Supplementary Table 4).

### A support vector machine accurately classifies progeroid diseases

A major hurdle for rare diseases is the possibility of fast diagnosis. We therefore generated a support vector machine (SVM) classifier that can identify premature aging diseases based on their phenotype alone. The SVM was trained to recognize progeroid syndromes by training it on the prevalence of phenotypes associated with the 32 progerias and to a control group of 29 non-progeroid syndromes from the database, which showed the least correlation with progeroid syndromes when using hierarchical clustering. The control syndromes are all non-mitochondrial syndromes with known pathogenesis. This yielded good results and the SVM accurately separates both progeroid and non-progeroid diseases in the database (Figure 2D). Notably, the SVM is available online at https://www.mitodb.com where inputted phenotypes will automatically receive an SVM score.

### Mitochondrial diseases show minor overlap to progeroid syndromes

**Figure 3:**
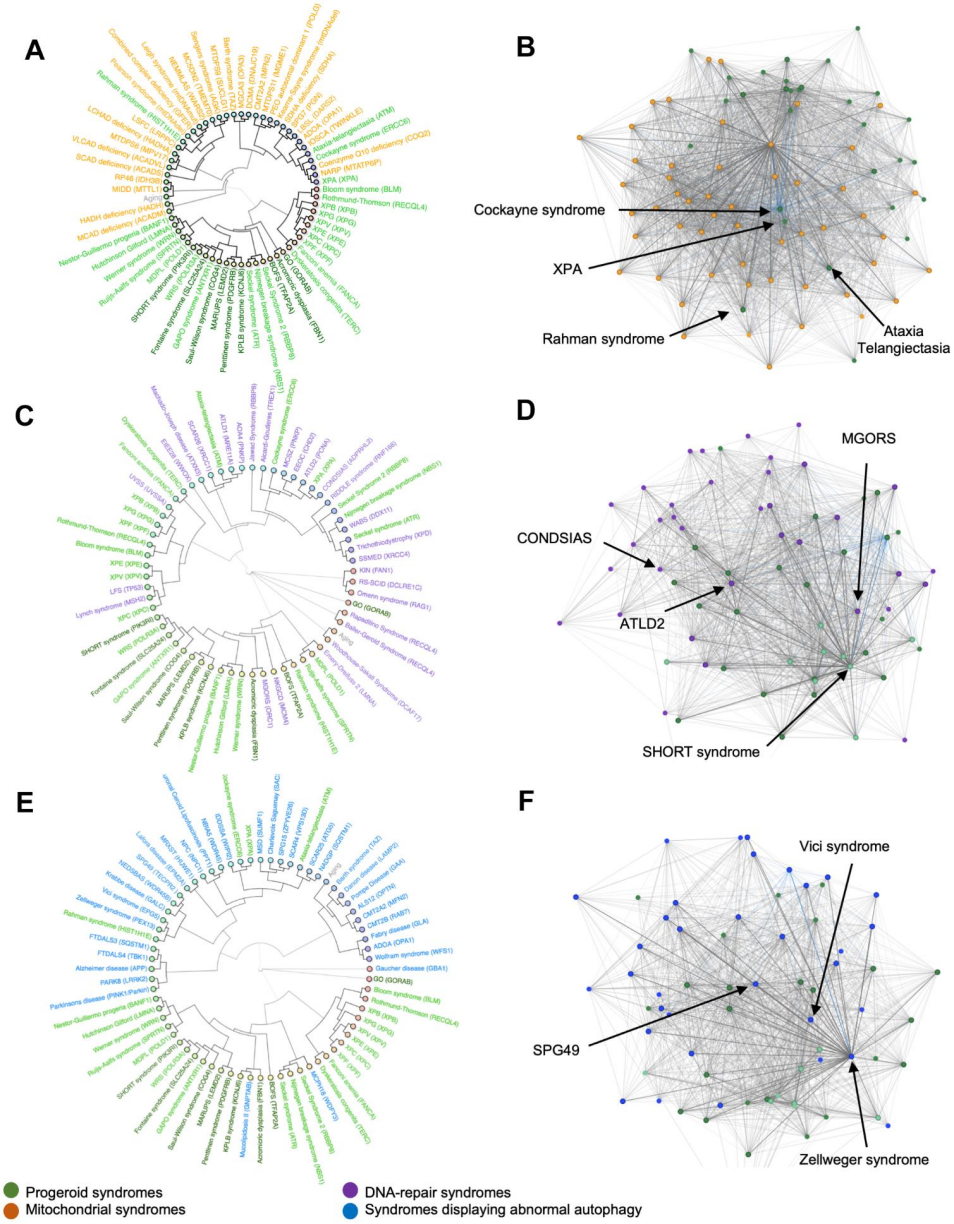
**Hierarchical clusters and syndrome networks.** Agglomerative hierarchical clustering and network algorithms of mitochondrial syndromes (**A**, **B**), DNA-repair syndromes (**C**, **D**) and syndromes with abnormal autophagy (**E**, **F**).

Mitochondrial alterations are known to occur with age and have been proposed to be a hallmark feature of aging. A connection has previously been established between mitochondrial syndromes and certain premature aging diseases such as Cockayne syndrome, xeroderma-pigmentosum and Ataxia-Telangiectasia [7, 9]. Therefore, we performed hierarchical clustering with the progeroid diseases and mitochondrial disorders (Figure 3A). Cockayne syndrome, xeroderma pigmentosum and Ataxia-Telangiectasia clustered with mitochondrial syndromes as expected. Notably, Rahman syndrome also clustered with mitochondrial syndromes suggesting phenotypic overlap. However, all other progeroid syndromes appeared to cluster exclusively with other progeroid syndromes.

To further investigate the link between mitochondrial and progeroid syndromes, we created a network to explore the connection between diseases. Each dot is a disease and a connecting line between dots represents a shared phenotype. The shorter the distance the more phenotypes are shared. This showed progeroid syndromes group in one end of the network indicating that they share significantly more traits among themselves even though they connect with mitochondrial syndromes (Figure 3B). Cockayne syndrome, Ataxia-Telangiectasia, Rahman syndrome and XPA were found scattered between the mitochondrial syndromes confirming their correlation as seen in the hierarchical cluster.

### DNA-repair syndromes show strong overlap with progerias

The progeroid syndromes in the data set are caused by mutations in single genes often associated with defect genome maintenance resulting in acceleration of some features in aging. Hutchinson-Gilford progeria, Cockayne syndrome and Werner syndrome are progeroid syndromes known to be caused by altered DNA-repair [10]. Creating a hierarchical cluster and a network for progeroid syndromes and DNA-repair syndromes allowed further analysis of their correlation. To investigate this, we clustered the premature aging diseases with diseases known for defects in DNA repair. As opposed to syndromes with mitochondrial pathogenesis, the DNA-repair syndromes were seen scattered between progeroid syndromes within the hierarchical cluster (Figure 3C). For instance, epileptic encephalopathy, childhood-onset (EEOC) and ataxia-telangiectasia like disorder 2 (ATLD2) clustered with Cockayne syndrome and XPA, Warsaw breakage syndrome (WABS) clustered with Seckel and Nijmegen breakage syndrome and Meier-Gorlin syndrome (MGORS) clustered with acromicric dysplasia.

Additionally, we created a network to compare with the hierarchical cluster. The network (Figure 3D) illustrated how certain progeroid syndromes such as SHORT syndrome were grouped in one end, suggesting that they share more traits among themselves than they do with other syndromes in the network. However, multiple DNA-repair syndromes such as ATLD2, neurodegeneration, childhood-onset, stress-induced, with variable ataxia and seizures (CONDSIAS) and MGORS were placed close to this group of progeroid syndromes indicating a large amount of shared traits and confirming our results from the hierarchical clustering.

### Syndromes characterized by abnormal autophagy show minor overlap with progerias

In healthy humans, the basal activity of autophagy in living cells decreases with age giving rise to accumulation of damaged cells. Notably, defective autophagy has recently been added as a hallmark of human aging [11]. To investigate the possible correlation between progeroid syndromes and autophagy we build a hierarchical cluster and a network comparing progeroid syndromes and syndromes displaying abnormal autophagy (Figure 3E, 3F). Surprisingly, the hierarchical clustering showed little clustering between progeroid syndromes and syndromes characterized by abnormal autophagy. Similarly, the hierarchical cluster with mitochondrial syndromes, Cockayne syndrome, XPA, Rahman syndrome and Ataxia Telangiectasia appeared to correlate phenotypically with syndromes presenting abnormal autophagy while the rest of the progeroid syndromes stayed clustered to each other. Notably defective mitophagy, a mitochondrial specific macro-autophagy pathway, has been shown for some premature aging disease [12]. The network complemented this result, showing progeroid syndromes distanced from the syndromes with abnormal autophagy. However, the syndromes spastic paraplegia 49 (SPG49), Vici syndrome and Zellweger syndrome were located close to the group of progeroid syndromes in the network making them potential candidates for further investigation.

### The premature aging phenome

**Figure 4:**
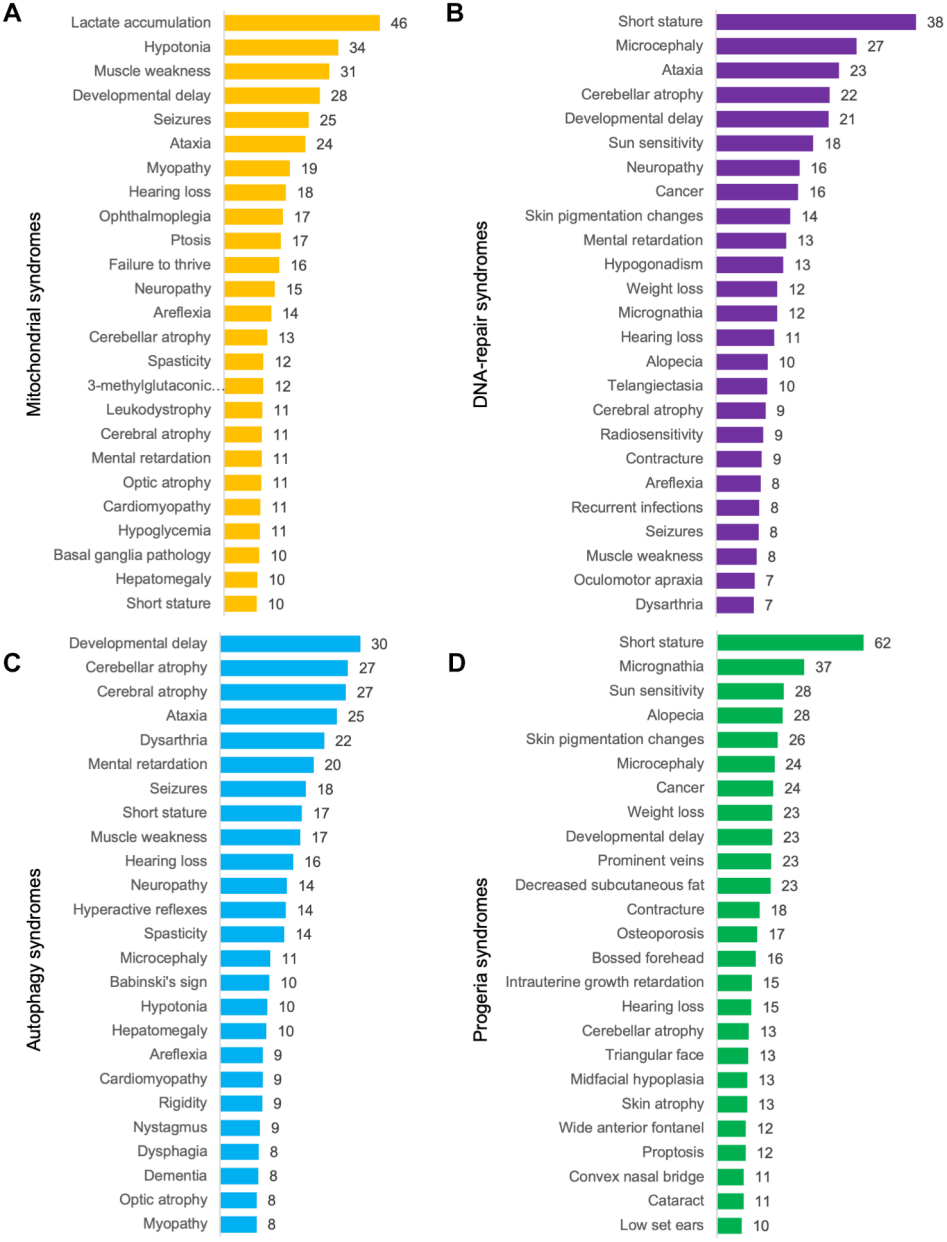
**Mean prevalence of phenotypes.** (**A**) Mitochondrial syndromes, (**B**) DNA-repair syndromes, (**C**) syndromes with abnormal autophagy and (**D**) progeroid syndromes.

Finally, we calculated the mean prevalence of phenotypes in each group of syndromes. Short stature, micrognathia, sun sensitivity, alopecia, skin pigmentation changes and microcephaly were discovered as being the most common phenotypes in progeroid syndromes (Figure 4). This showed considerable overlap with diseases with abnormal DNA-repair where short stature, microcephaly, ataxia, cerebellar atrophy and developmental delay were the most common phenotypes. In mitochondrial syndromes lactate accumulation, hypotonia, developmental delay, muscle weakness, seizures and ataxia were the most common phenotypes [7]. Lastly, developmental delay, cerebellar and cerebral atrophy, ataxia and dysarthria were the most common phenotypes in syndromes with abnormal autophagy perhaps illustrating that defective authophagy can lead to mitochondrial dysfunction. In conclusion, progeroid syndromes therefore seem to share most phenotypes with DNA-repair syndromes as shown previously in our hierarchical cluster and networks.

### Identification of possible progeroid syndromes

In order to find potential progeroid syndromes amongst the mitochondrial syndromes, DNA-repair syndromes and syndromes with abnormal autophagy, we applied a high threshold when working with networks (see Methods). The majority of these syndromes were not associated with progeroid syndromes when the threshold was raised and were mainly connected to others of the same classification. However, three syndromes were more strongly linked to progeroid syndromes in their respective networks, suggesting a correlation of phenotypic traits. They included ATLD2 (Ataxia Telangiectasia like Disorder 2) and MGORS (Meier-Gorlin syndrome) that are both DNA-repair syndromes and SPG49 (Spastic Paraplegia 49), a syndrome displaying abnormal autophagy.

**Figure 5:**
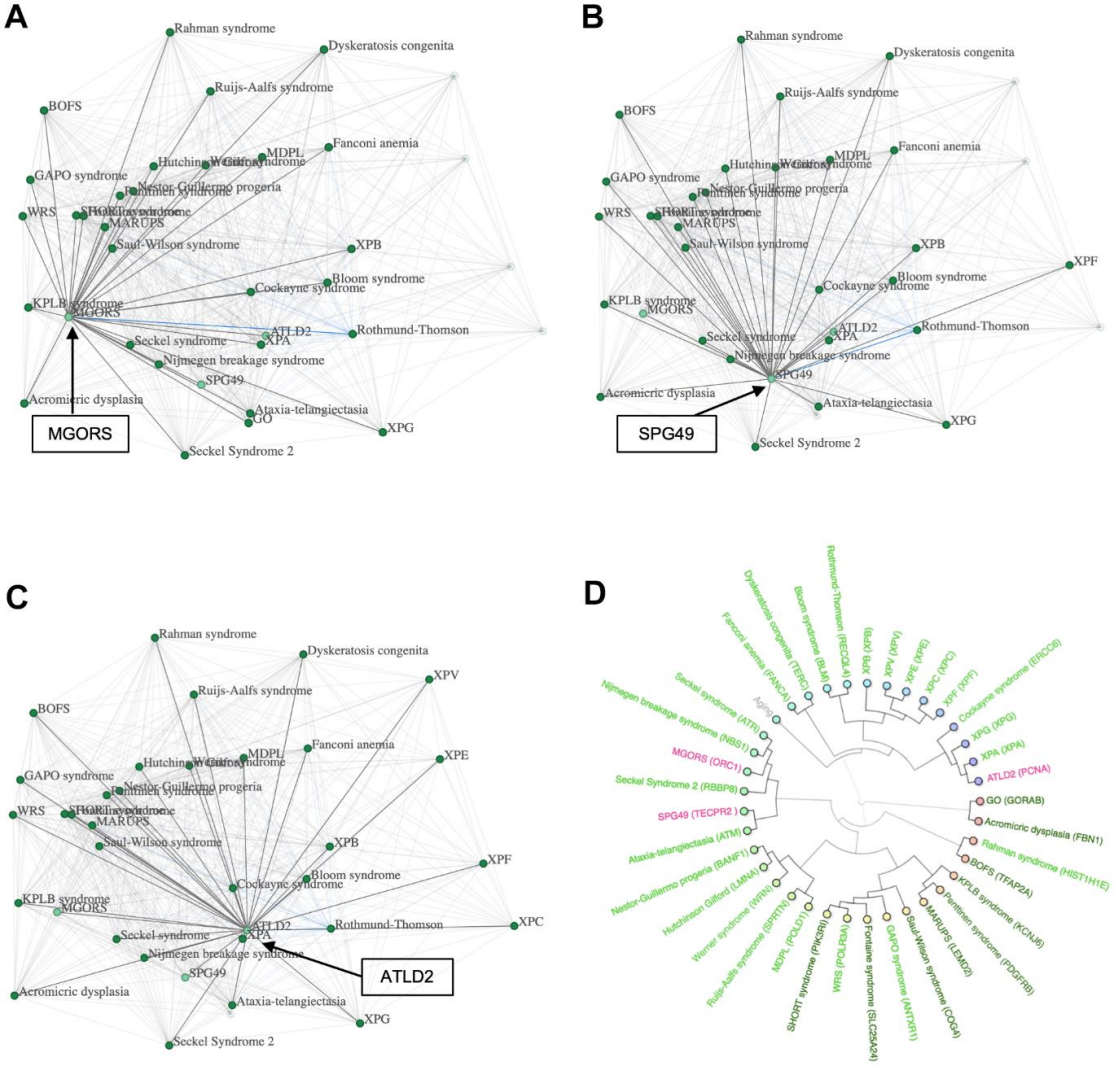
**Possible progeroid syndromes.** (**A**) A network containing progeroid syndromes and MGORS, (**B**) network containing progeroid syndromes and SPG49, (**C**) network containing progeroid syndromes and ATDL2 and (**D**) a hierarchical cluster with progeroid syndromes, MGORS, ATLD2 and SPG49.

To test their potential to be progeroid, we incorporated them in a network with all progeroid syndromes (Figure 5A–5C) with the aim of investigating how the addition of the syndromes would alter the network. Firstly, we tested our network exclusively containing progeroid syndromes by raising the threshold, which led to the syndromes GO, XPC, XPF and XPV to disconnect from the rest. After adding the three syndromes ATLD2, SPG49 and MGORS the same four progeroid syndromes disconnected from the network, indicating a strong connection between the three tested syndromes and the main cluster of progeroid syndromes. Furthermore, when looking at the position of syndromes within the networks there seemed to be few changes. All tested syndromes were located near the center group of progeroid syndromes. This indicated that the incorporated syndromes have similar phenotypic profiles and do not result in change in the correlation between known progeroid syndromes.

To further investigate this hypothesis, we created a hierarchical cluster containing the three tested syndromes and all progeroid syndromes known to the database (Figure 5D). The known progeroid syndromes clustered in their usual groups as previously visualized in Figure 2A, showing little interference when adding the three new syndromes. ATLD2 clustered with the group of progeroid syndromes containing Xeroderma Pigmentosum A and Cockayne syndrome, SPG49 clustered with Ataxia Telangiectasia, and MGORS clustered with Seckel syndrome and Nijmegen breakage syndrome. Lastly, we used the SVM to predict the syndromes’ likelihood of being progeroid. ATLD2 scored 3.49, SPG49 scored 1.75 and MGORS scored 1.56 supporting the hypothesis of these syndromes being progeroid.

To further illustrate the predictive power of our algorithms we further decided to test if Charcot-Marie-Tooth disease (CMT2Z), a disease recently associated with Cockayne Syndrome [13], could be a possible new progeria. Hierarchical clustering indeed showed close phenotypical correlation with Cockayne syndrome (Supplementary Figure 3A) and a relatively high SVM score of 1.24 suggesting that the disorder could be classified as progeroid.

## DISCUSSION

In this study, we analyzed the prevalence of phenotypes observed in progeroid syndromes to define the progeroid phenome. This allowed us to identify the combination of traits which presented with the highest prevalence in progeroid syndromes. The phenotypes with the highest prevalence included short stature, micrognathia, sun sensitivity, alopecia, skin pigmentation changes, microcephaly and cancer. The full set of phenotypes create the foundation for the ‘progeria phenome’, which can be used for comparisons and diagnostics in the future and is accessible on https://www.mitodb.com. Of note, anyone can test if a patient or disease display phenotypical overlap with these diseases by inputting features on the website and test the disease.

To better understand the correlation between progeroid syndromes internally, we modulated the data sets through networks and hierarchical clustering. The progeroid syndromes seemed to cluster and network in groups indicating a certain variety within progerias. The syndromes SHORT, Saul-Wilson, Penttinen, Fontaine, WRS, GAPO and KPLB presented with close correlation independent of which category of syndromes they were compared to throughout the study. Interestingly, gene mutation in each of these syndromes have been linked to defects in cell differentiation and proliferation suggesting common pathogenesiss [14–19]. Several other groupings repeatedly clustered together in hierarchical clustering and networks, indicating the presence of subgroups within the progeroid syndromes. Accordingly, some progeroid syndromes have been linked with mitochondrial syndromes, DNA-repair syndromes and syndromes displaying abnormal autophagy. However, we saw weak correlation between progerias and either mitochondrial syndromes or syndromes displaying abnormal autophagy as opposed to progeroid and DNA-repair syndromes with which there was a clear correlation. This suggests that the pathogenesis of progeroid syndromes is related to DNA-repair syndromes or that the gestalt of progeroid syndromes is more in line with aging, while autophagy and mitochondrial disease perhaps show aging phenotypes linked with internal organs such as the brain.

Our transcriptomics analysis of premature aging diseases provides further understanding of expression signatures within analogous disease subsets. We observe that in some diseases, the gene expression-based clustering demonstrates consistency with phenotypic clustering, however, this is not the case for all diseases. Notably, specific disorders such as Cockayne syndrome, Xeroderma Pigmentosum A (XPA), Ataxia-Telangiectasia, Werner syndrome, and Hutchinson Gilford exhibited a tendency to cluster based on the congruity of their gene expression patterns. However, it should be noted that certain significant discrepancies reveal that occasionally, the distinctions between the molecular mechanisms of a disease may not be accurately captured by phenotypic information alone. For instance, we identified divergences in clustering patterns, as evidenced by Bloom syndrome clustering with Nijmegen breakage syndrome and Seckel syndrome clustering in conjunction with Nestor-Guillermo progeria syndrome.

Furthermore, the hierarchical clusterings and networks led to the discovery of possible new progeroid syndromes. Focusing on the networks we identified tendencies in three non-progeroid syndromes, ATLD2, MGORS and SPG49, suggesting that they had similar clinical traits as those of progeroid syndromes. However, this assumption seemed imprecise when using hierarchical clustering as only MGORS had significant clusters to progeroid syndromes. Nevertheless, it is important to differentiate between hierarchical clustering and networks, as hierarchical clustering forces syndromes into clusters solely based on the level of correlation. Consequently, a syndrome will be forced in a cluster containing the specific syndrome with which it shares the most traits. In contrast, a syndrome maintains links to all other syndromes they share phenotypes with in a network. The location of a syndrome is based on the amount it correlates with others, placing syndromes close if they share many traits and further apart if they only share a few. Therefore, we maintain our suggestion that the three diseases could possibly be classified as progeroid syndromes.

As progeroid syndromes have historically been diagnosed and described based solely on phenotypes, comparing syndromes’ phenotypes to the progeroid phenome using hierarchical clusterings, networks and mean prevalence is a useful and reliable tool for the potential identification of new progeroid syndromes. Further, tools such as this can identify patterns not previously recognized in known progerias, for instance we observe that XPA, CS and AT cluster closely together while XPB [20] and XPG [21] that in rare cases can lead to a CS like phenotype are in a separate, but associated, cluster. This could be explained by the rarity of the XPG and XPB-CS patients leading to only minor contributions to the overall XPB and XPG phenotype. Creating a machine learning algorithm based on the progeria phenome, giving the tested syndrome a score based on its likelihood of being progeroid, could be an opportunity to optimize the, often slow [22] diagnostic process for these disorders. However, as phenotypes are predominantly a subjective parameter relying on the observing physician and their focus, we argue that having objective tools for diagnosing and analyzing progeroid syndromes would further strengthen the process. This may aid in other diagnostic tools such as whole genome or exome sequencing. Consequently, having objective parameters for progeroid syndromes would strengthen the diagnosis of patients as well as create a more detailed picture of a syndrome and its further progression.

In conclusion, we can analyze and evaluate a syndrome’s likelihood of being progeroid by using hierarchical clustering, networks and the mean prevalence of phenotypes seen in known progeroid syndromes. Additionally, defining the progeria phenome optimizes clinical diagnosis of patients presenting with a variety of phenotypes and has allowed us to develop a support vector machine that can predict a syndrome’s likelihood of being progeroid solely based on phenotypes. Notably, this can already be explored on https://www.mitodb.com. The prospect of identifying clear phenotypic profiles and comparing objective parameters such as pathogenesis, biochemical markers and physiological markers can serve as the basis for early diagnosis, improved patient treatment, and the hope of developing innovative medicines.

## MATERIALS AND METHODS

### OMIM.org

This study is a literature review based on the selection of relevant articles focusing on reviews, meta-analysis and clinical studies. The primary source of articles was PubMed Central (PMC) and the process of selecting articles occurred October 1st - November 5th. When designing our search, we used the website https://www.OMIM.org as a base. OMIM.org is an online compendium of the human genes and genetic phenotypes. The site contains PMC referenced full-text overviews on all known Mendelian disorders and over 16.000 genes. OMIM additionally has a unique search feature facilitating the option of focusing the search on diseases with phenotype description and known molecular basis.

The literature search was based on an “Advanced Search” using relevant medical subject heading and keywords. The relevant terms and keywords were then combined by exploiting the Boolean Operators ‘OR’, ‘AND’, ‘NOT’. The process of specifying the search originated from the terms ‘Progeriod’ and ‘Progeria’ with opt in ‘# phenotype description, molecular basis known’ due to the purpose being phenotypic analysis. This search was revealed to be insufficient, seeing as the result excluded several confirmed progeroid syndromes. Therefore, we added ‘premature aging’ in addition to the original terms as keywords with a proximity search to limit the distance in words between two keywords to 1. The second search resulted in 66 hits including all previously reported as well as unreported progeroid syndromes on https://www.mitodb.com, making this the base of further selection.

To reinforce the quality of the study we supplemented the OMIM search with an additional PMC search focused on articles published later than those referenced on OMIM. This thoroughness ensured that using OMIM as the original source of literature did not lead to insufficient or inadequate literature for the study. As an example, the Brachiooculofacial syndrome had 24 articles referenced on OMIM, the latest dating back to 2015. To account for research made after 2015, we supplemented with an additional PMC search using the keyword “branchiooculofacial” and focusing the search on articles published from Jan 1st 2015 and forward. This resulted in 7 hits, giving us a total of 31 articles on the syndrome. After screening all articles, 24 were excluded on the basis of the exclusion criteria, thereby limiting the result to accessible English articles describing the syndromes phenotype in 2 or more unrelated individuals. The articles excluded were case reports, articles without phenotypic description, non-English articles and articles without full text link. As illustrated in Figure 1A, an identical processing was performed for all 9 syndromes leading to a total of 26 articles [14, 17, 23–46].

### Inclusion and exclusion

Originally, we included 19 syndromes (see Figure 1) since they all presented with phenotypic traits of progeroid syndromes and had monogenic originance therefore fulfilling our inclusion criteria. However, to ensure the quality of the study we excluded 10 disorders presenting with only 1 studied patient or based purely on biologically related patients. Other syndromes already existed in the database and therefore, naturally, were excluded. The remaining 9 mendelian disorders met the inclusion criteria by respectively being new to the database, showing phenotypic signs of progeria, having a monogenic origin and having been studied in two or more unrelated patients.

### Hierarchical clustering

Hierarchical clustering illustrates how diseases cluster with each other based on correlation of their traits by creating a dendrogram [47]. The dendrogram connects diseases sharing similar traits by measuring similarity and linkage methods. In the dendrogram two closely correlated diseases will be portrayed with a short distance and short leg. Furthermore, the tool calculates a cophenetic correlation when clustering diseases to ensure resemblance of the original sample distance and the cophenetic distance in the dendrogram. The value measures how well the clustering result matches the original resemblance by identifying what uncentered similarity and average linkage will produce the best correlation [48].

### The disease network

The Disease Network is a network connecting syndromes by shared phenotypic traits. Each dot represents a syndrome and each line between dots represents a shared trait. If multiple traits are shared by two syndromes, the line will appear darker, thicker and shorter. It is possible to apply different thresholds in order to limit the network so that it only displays diseases with a significant number of shared traits. Some features occur in almost all patients and are therefore perhaps more biologically relevant for the particular disease. However, patients may also suffer from features that are not seen in all patients and therefore are not as biologically relevant. By applying a threshold where only high prevalence features are considered, less common features are excluded. Thresholding is performed by summing the product of percentage prevalence for each shared phenotype. For instance, if two diseases share one phenotype (e.g. short stature) with 10% each, the product score is 100. The same product score would be calculated in the case for 1% in one disease and 100% percent in another disease. However, if one disease has 100% and another 100% then the product score is 10.000. The product score is thus exponentially higher for diseases with higher percentage phenotypic overlap. If there are more than one shared phenotype then the product score for each phenotype is summed. The lowest threshold is a product score of 1000 which indicates at least an overlap of at least 34% in at least one phenotype while the highest threshold currently available is 20.000 which indicates at least two shared phenotypes with 100% prevalence in both diseases.

### Mean prevalence of phenotypes

The mean prevalence of phenotypes was calculated from manually curated literature where each phenotype was mentioned. We calculated the mean prevalence of phenotypes for a disease by calculating the sum of all individuals with the phenotype and divide it with all individuals examined for that phenotype. The prevalence of phenotypes for disease categories is the mean prevalence for each phenotype in each disease in that category.

### Principal component analysis

Principal component analysis was performed on the disease and phenotype matrix using cluster 3.0 [49].

### Support vector machine

The support vector machine (SVM) was created by exporting the symptom-vectors of the known progeria and non-progeria diseases. The symptom-vectors were processed in python using the ‘SVC’ class from scikit-learn to generate an SVM. The SVM was trained using a “linear kernel”. The parameters of the SVM were then exported and the classifier functions for the web-page was created (with php) using these parameters [50, 51].

### Omics data collection and hierarchical clustering

Publicly available gene expression data for 13 progeroid syndromes were collected using PandaOmics [52], an AI-driven target discovery platform with its proprietary pathway analysis approach, iPANDA [53]. The syndromes included Branchio-Oculo-Facial Syndrome (GSE108521 (RNA-seq, Neural crest cell line)), Ataxia-telangiectasia (GSE75852 (RNA-seq, NPCs), E-MTAB-1217 (Microarray, NPCs), GSE142842 (RNA-seq, blood), GSE61019 (Microarray, cortex), GSE152287 (Microarray, fibroblasts), GSE35347 (Microarray, fibroblasts), GSE35347 (Microarray, IPSCs), GSE75852 (RNA-seq, IPSCs)), Bloom Syndrome (GSE54502 (Microarray, fibroblasts), GSE123447 (Microarray, fibroblasts)), Cockayne syndrome (GSE144557 (Microarray, Cerebellum), GSE36648 (Microarray, IPSCs)), Dyskeratosis congenita (GSE64023 (Microarray, MSCs), GSE77525 (Microarray, T cells), GSE83501 (RNA-seq, lung)), Hutchinson-Gilford progeria syndrome (E-MEXP-2597 (Microarray, fibroblasts), GSE28863 (Microarray, fibroblasts), GSE113957 (RNA-seq, fibroblasts), GSE137083 (RNA-seq, fibroblasts), GSE3860 (Microarray, fibroblasts)), Nestor-Guillermo progeria syndrome (GSE65170 (Microarray, fibroblast), GSE65172 (Microarray, Mesenchymal stem cell)), Nijmegen breakage Syndrome (GSE83686 (Microarray, NPCs), GSE94707 (Microarray, fibroblasts), GSE13909 (Microarray, lymphoblasts)), Seckel syndrome (GSE121384 (RNA-seq, IPSCs)), Werner Syndrome (GSE48761 (Microarray, fibroblasts)), Xeroderma Pigmentosum group A (GSE55484 (Microarray, fibroblasts)), Xeroderma Pigmentosum group C (GSE119501 (RNA-seq, fibroblasts), GSE133084 (Microarray, fibroblasts)), and Xeroderma Pigmentosum group V (GSE70818 (Microarray, fibroblasts)).

All gene expression datasets were processed according to platform-specific protocols. Differential expression analysis was performed using the limma package. For each disease, the results of gene expression comparisons were combined into a meta-analysis, which was utilized to identify disease expression signatures. Using PandaOmics, disease-expression signatures were extracted from the meta-analysis section, which allowed users to calculate logarithmic fold-changes (LFCs) and Q-values across all gene expression datasets. This was achieved using min-max normalization for LFC values and Stouffer’s method for combining p-values. Disease-expression signature vectors with combined LFC values were aggregated into a matrix, which was then used for hierarchical clustering analysis. Genes not expressed in all 13 progeroid syndromes were excluded from the expression table. Hierarchical clustering was performed using the scipy python package and the cluster.hierarchy.linkage function with metric=‘euclidean’ and method=‘average’. The hierarchical clustering results were visualized using the ete3 python package Tree, TreeStyle, NodeStyle, and TextFace functions.

### Signaling pathway analysis

Pathway analysis for 13 progeroid syndromes was performed using iPANDA algorithm [53]. Reactome signaling pathway graph was used as a database for the iPANDA algorithm [54]. Using a linear combination of logarithmic fold-changes, statistical weights, and topological weights applied to each pathway member gene, iPANDA algorithm estimates the direction and intensity of pathway activation. Accordingly, the output of iPANDA represents the difference in gene expression between disease and control groups, and the iPANDA score is used to calculate the score for each signaling pathway. A high iPANDA score indicates an upregulation of a pathway, whereas a low score indicates a downregulation. Combined iPANDA scores for each disease were calculated using min-max normalization for iPANDA values and Stouffer’s method for combining p-values. Lists with significantly (Combined iPANDA p-value < 0.05) positively upregulated and downregulated pathways were collected and overlaps between groups of progeroid syndromes (Group 1 includes Xeroderma Pigmentosum group A, Cockayne syndrome, and Ataxia-telangiectasia, Group 2 includes Werner syndrome and Hutchinson-Gilford progeria, Group 3 includes Bloom syndrome and Nijmegen breakage, Group 4 includes Nestor-Guillermo and Seckel; Group 5 includes Hutchinson-Gilford progeria, Werner syndrome and Nestor-Guillermo; Group 6 includes Bloom syndrome, Xeroderma Pigmentosum group C and Xeroderma Pigmentosum group V) were performed and visualised using *upsetplot* python package.

## Supplementary Material

Supplementary Figures

Supplementary Table 1

Supplementary Table 2

Supplementary Table 3

Supplementary Table 4

## References

[1] Kaposi K, Fagge HC, Hebra F. On Diseases of the Skin, Including the Exanthemata. 2022. https://archive.org/details/ondiseasesskini00hebrgoog/page/n274/mode/2up

[2] Hutchinson J. Congenital Absence of Hair and Mammary Glands with Atrophic Condition of the Skin and its Appendages, in a Boy whose Mother had been almost wholly Bald from Alopecia Areata from the age of Six. Med Chir Trans. 1886; 69:473–7. 10.1177/095952878606900127 20896687 PMC2121576

[3] López-Otín C, Blasco MA, Partridge L, Serrano M, Kroemer G. Hallmarks of aging: An expanding universe. Cell. 2023; 186:243–78. 10.1016/j.cell.2022.11.001 36599349

[4] Petr MA, Tulika T, Carmona-Marin LM, Scheibye-Knudsen M. Protecting the Aging Genome. Trends Cell Biol. 2020; 30:117–32. 10.1016/j.tcb.2019.12.001 31917080

[5] Capell BC, Tlougan BE, Orlow SJ. From the rarest to the most common: insights from progeroid syndromes into skin cancer and aging. J Invest Dermatol. 2009; 129:2340–50. 10.1038/jid.2009.103 19387478

[6] Frésard L, Montgomery SB. Diagnosing rare diseases after the exome. Cold Spring Harb Mol Case Stud. 2018; 4:a003392. 10.1101/mcs.a003392 30559314 PMC6318767

[7] Scheibye-Knudsen M, Scheibye-Alsing K, Canugovi C, Croteau DL, Bohr VA. A novel diagnostic tool reveals mitochondrial pathology in human diseases and aging. Aging (Albany NY). 2013; 5:192–208. 10.18632/aging.100546 23524341 PMC3629291

[8] Andreassen SN, Ben Ezra M, Scheibye-Knudsen M. A defined human aging phenome. Aging (Albany NY). 2019; 11:5786–806. 10.18632/aging.102166 31408848 PMC11627290

[9] Scheibye-Knudsen M, Tseng A, Borch Jensen M, Scheibye-Alsing K, Fang EF, Iyama T, Bharti SK, Marosi K, Froetscher L, Kassahun H, Eckley DM, Maul RW, Bastian P, et al. Cockayne syndrome group A and B proteins converge on transcription-linked resolution of non-B DNA. Proc Natl Acad Sci USA. 2016; 113:12502–7. 10.1073/pnas.1610198113 27791127 PMC5098674

[10] Kyng KJ, Bohr VA. Gene expression and DNA repair in progeroid syndromes and human aging. Ageing Res Rev. 2005; 4:579–602. 10.1016/j.arr.2005.06.008 16246641

[11] Aman Y, Schmauck-Medina T, Hansen M, Morimoto RI, Simon AK, Bjedov I, Palikaras K, Simonsen A, Johansen T, Tavernarakis N, Rubinsztein DC, Partridge L, Kroemer G, et al. Autophagy in healthy aging and disease. Nat Aging. 2021; 1:634–50. 10.1038/s43587-021-00098-4 34901876 PMC8659158

[12] Bakula D, Scheibye-Knudsen M. MitophAging: Mitophagy in Aging and Disease. Front Cell Dev Biol. 2020; 8:239. 10.3389/fcell.2020.00239 32373609 PMC7179682

[13] Stafki SA, Turner J, Littel HR, Bruels CC, Truong D, Knirsch U, Stettner GM, Graf U, Berger W, Kinali M, Jungbluth H, Pacak CA, Hughes J, et al. The Spectrum of MORC2-Related Disorders: A Potential Link to Cockayne Syndrome. Pediatr Neurol. 2023; 141:79–86. 10.1016/j.pediatrneurol.2023.01.011 36791574 PMC10098370

[14] Writzl K, Maver A, Kovačič L, Martinez-Valero P, Contreras L, Satrustegui J, Castori M, Faivre L, Lapunzina P, van Kuilenburg ABP, Radović S, Thauvin-Robinet C, Peterlin B, et al. De Novo Mutations in SLC25A24 Cause a Disorder Characterized by Early Aging, Bone Dysplasia, Characteristic Face, and Early Demise. Am J Hum Genet. 2017; 101:844–55. 10.1016/j.ajhg.2017.09.017 29100094 PMC5673633

[15] Gene Cards 2022. “PDGFRB Gene - Platelet Derived Growth Factor Receptor Bet”. genecards.org. 2022. https://www.genecards.org/cgi-bin/carddisp.pl?gene=PDGFRB

[16] Gene Cards 2022. “LEMD2 Gene - LEM Domain Nuclear Envelope Protein 2”. genecards.org. 2022. https://www.genecards.org/cgi-bin/carddisp.pl?gene=LEMD2

[17] Masotti A, Uva P, Davis-Keppen L, Basel-Vanagaite L, Cohen L, Pisaneschi E, Celluzzi A, Bencivenga P, Fang M, Tian M, Xu X, Cappa M, Dallapiccola B. Keppen-Lubinsky syndrome is caused by mutations in the inwardly rectifying K+ channel encoded by KCNJ6. Am J Hum Genet. 2015; 96:295–300. 10.1016/j.ajhg.2014.12.011 25620207 PMC4320262

[18] Smaldone S, Clayton NP, del Solar M, Pascual G, Cheng SH, Wentworth BM, Schaffler MB, Ramirez F. Fibrillin-1 Regulates Skeletal Stem Cell Differentiation by Modulating TGFβ Activity Within the Marrow Niche. J Bone Miner Res. 2016; 31:86–97. 10.1002/jbmr.2598 26189658 PMC5776390

[19] Gene Cards 2022. “PIK3R1 Gene - Phosphoinositide-3-Kinase Regulatory Subunit 1”. genecards.org. 2022. https://www.genecards.org/cgi-bin/carddisp.pl?gene=PIK3R1

[20] Oh KS, Khan SG, Jaspers NG, Raams A, Ueda T, Lehmann A, Friedmann PS, Emmert S, Gratchev A, Lachlan K, Lucassan A, Baker CC, Kraemer KH. Phenotypic heterogeneity in the XPB DNA helicase gene (ERCC3): xeroderma pigmentosum without and with Cockayne syndrome. Hum Mutat. 2006; 27:1092–103. 10.1002/humu.20392 16947863

[21] Emmert S, Slor H, Busch DB, Batko S, Albert RB, Coleman D, Khan SG, Abu-Libdeh B, DiGiovanna JJ, Cunningham BB, Lee MM, Crollick J, Inui H, et al. Relationship of neurologic degeneration to genotype in three xeroderma pigmentosum group G patients. J Invest Dermatol. 2002; 118:972–82. 10.1046/j.1523-1747.2002.01782.x 12060391

[22] Schnabel F, Kornak U, Wollnik B. Premature aging disorders: A clinical and genetic compendium. Clin Genet. 2021; 99:3–28. 10.1111/cge.13837 32860237

[23] Maroteaux P, Stanescu R, Stanescu V, Rappaport R. Acromicric dysplasia. Am J Med Genet. 1986; 24:447–59. 10.1002/ajmg.1320240307 3728563

[24] Faivre L, Le Merrer M, Baumann C, Polak M, Chatelain P, Sulmont V, Cousin J, Bost M, Cordier MP, Zackai E, Russell K, Finidori G, Pouliquen JC, et al. Acromicric dysplasia: long term outcome and evidence of autosomal dominant inheritance. J Med Genet. 2001; 38:745–9. 10.1136/jmg.38.11.745 11694546 PMC1734753

[25] Sato T, Samura O, Kato N, Taniguchi K, Takahashi K, Ito Y, Aoki H, Kobayashi M, Migita O, Okamoto A, Hata K. Novel TFAP2A mutation in a Japanese family with Branchio-oculo-facial syndrome. Hum Genome Var. 2018; 5:5. 10.1038/s41439-018-0004-z 29760939 PMC5945586

[26] Stoetzel C, Riehm S, Bennouna Greene V, Pelletier V, Vigneron J, Leheup B, Marion V, Hellé S, Danse JM, Thibault C, Moulinier L, Veillon F, Dollfus H. Confirmation of TFAP2A gene involvement in branchio-oculo-facial syndrome (BOFS) and report of temporal bone anomalies. Am J Med Genet A. 2009; 149:2141–6. 10.1002/ajmg.a.33015 19764023

[27] Lee WK, Root AW, Fenske N. Bilateral branchial cleft sinuses associated with intrauterine and postnatal growth retardation, premature aging, and unusual facial appearance: a new syndrome with dominant transmission. Am J Med Genet. 1982; 11:345–52. 10.1002/ajmg.1320110311 7200726

[28] Richardson E, Davison C, Moore AT. Colobomatous microphthalmia with midfacial clefting: part of the spectrum of branchio-oculo-facial syndrome? Ophthalmic Genet. 1996; 17:59–65. 10.3109/13816819609057872 8832722

[29] Lam K, Cassidy B, Arreola R, Al Saif H, King K, Couser NL. A New Case and Comprehensive Review of the Ophthalmic Manifestations of 172 Individuals With Branchio-Oculo-Facial Syndrome. J Pediatr Ophthalmol Strabismus. 2023; 60:295–301. 10.3928/01913913-20220825-01 36263936

[30] Lin AE, Gorlin RJ, Lurie IW, Brunner HG, van der Burgt I, Naumchik IV, Rumyantseva NV, Stengel-Rutkowski S, Rosenbaum K, Meinecke P. Further delineation of the branchio-oculo-facial syndrome. Am J Med Genet. 1995; 56:42–59. 10.1002/ajmg.1320560112 7747785

[31] Titheradge HL, Patel C, Ragge NK. Branchio-oculo-facial syndrome: a three generational family with markedly variable phenotype including neonatal lethality. Clin Dysmorphol. 2015; 24:13–6. 10.1097/MCD.0000000000000056 25325185

[32] Bayram Y, Pehlivan D, Karaca E, Gambin T, Jhangiani SN, Erdin S, Gonzaga-Jauregui C, Wiszniewski W, Muzny D, Elcioglu NH, Yildirim MS, Bozkurt B, Zamani AG, et al, and Baylor-Hopkins Center for Mendelian Genomics. Whole exome sequencing identifies three novel mutations in ANTXR1 in families with GAPO syndrome. Am J Med Genet A. 2014; 164:2328–34. 10.1002/ajmg.a.36678 25045128 PMC4332576

[33] Avila M, Dyment DA, Sagen JV, St-Onge J, Moog U, Chung BHY, Mo S, Mansour S, Albanese A, Garcia S, Martin DO, Lopez AA, Claudi T, et al. Clinical reappraisal of SHORT syndrome with PIK3R1 mutations: toward recommendation for molecular testing and management. Clin Genet. 2016; 89:501–6. 10.1111/cge.12688 26497935

[34] Lisker R, Hernández A, Martínez-Lavin M, Mutchinick O, Armas C, Reyes P, Robles-Gil J. Gerodermia osteodysplastica hereditaria: report of three affected brothers and literature review. Am J Med Genet. 1979; 3:389–95. 10.1002/ajmg.1320030410 474638

[35] al-Torki NA, al-Awadi SA, Cindro-Heberie L, Sabry MA. Gerodermia osteodysplastica in a Bedouin sibship: further delineation of the syndrome. Clin Dysmorphol. 1997; 6:51–5. 10.1097/00019605-199701000-00009 9018419

[36] Hunter AG, Martsolf JT, Baker CG, Reed MH. Geroderma osteodysplastica. A report of two affected families. Hum Genet. 1978; 40:311–24. 10.1007/BF00272192 631850

[37] Zufferey F, Hadj-Rabia S, De Sandre-Giovannoli A, Dufier JL, Leheup B, Schweitze C, Bodemer C, Cormier-Daire V, Le Merrer M. Acro-osteolysis, keloid like-lesions, distinctive facial features, and overgrowth: two newly recognized patients with premature aging syndrome, Penttinen type. Am J Med Genet A. 2013; 161:1786–91. 10.1002/ajmg.a.35984 23720404

[38] Johnston JJ, Sanchez-Contreras MY, Keppler-Noreuil KM, Sapp J, Crenshaw M, Finch NA, Cormier-Daire V, Rademakers R, Sybert VP, Biesecker LG. A Point Mutation in PDGFRB Causes Autosomal-Dominant Penttinen Syndrome. Am J Hum Genet. 2015; 97:465–74. 10.1016/j.ajhg.2015.07.009 26279204 PMC4564935

[39] Bredrup C, Stokowy T, McGaughran J, Lee S, Sapkota D, Cristea I, Xu L, Tveit KS, Høvding G, Steen VM, Rødahl E, Bruland O, Houge G. A tyrosine kinase-activating variant Asn666Ser in PDGFRB causes a progeria-like condition in the severe end of Penttinen syndrome. Eur J Hum Genet. 2019; 27:574–81. 10.1038/s41431-018-0323-z 30573803 PMC6460636

[40] Gorlin RJ, Chaudhry AP, Moss ML. Craniofacial dysostosis, patent ductus arteriosus, hypertrichosis, hypoplasia of labia majora, dental and eye anomalies-a new syndrome? J Pediatr. 1960; 56:778–85. 10.1016/s0022-3476(60)80315-0 13851313

[41] Petty EM, Laxova R, Wiedemann HR. Previously unrecognized congenital progeroid disorder. Am J Med Genet. 1990; 35:383–7. 10.1002/ajmg.1320350314 2309786

[42] Ehmke N, Graul-Neumann L, Smorag L, Koenig R, Segebrecht L, Magoulas P, Scaglia F, Kilic E, Hennig AF, Adolphs N, Saha N, Fauler B, Kalscheuer VM, et al. De Novo Mutations in SLC25A24 Cause a Craniosynostosis Syndrome with Hypertrichosis, Progeroid Appearance, and Mitochondrial Dysfunction. Am J Hum Genet. 2017; 101:833–43. 10.1016/j.ajhg.2017.09.016 29100093 PMC5673623

[43] Saul RA, Wilson WG. A “new” skeletal dysplasia in two unrelated boys. Am J Med Genet. 1990; 35:388–93. 10.1002/ajmg.1320350315 2309787

[44] Ferreira CR, Xia ZJ, Clément A, Parry DA, Davids M, Taylan F, Sharma P, Turgeon CT, Blanco-Sánchez B, Ng BG, Logan CV, Wolfe LA, Solomon BD, et al, Undiagnosed Diseases Network, and Scottish Genome Partnership. A Recurrent De Novo Heterozygous COG4 Substitution Leads to Saul-Wilson Syndrome, Disrupted Vesicular Trafficking, and Altered Proteoglycan Glycosylation. Am J Hum Genet. 2018; 103:553–67. 10.1016/j.ajhg.2018.09.003 30290151 PMC6174323

[45] Hersh JH, Joyce MR, Spranger J, Goatley EC, Lachman RS, Bhatt S, Rimoin DL. Microcephalic osteodysplastic dysplasia. Am J Med Genet. 1994; 51:194–9. 10.1002/ajmg.1320510304 8074143

[46] Marbach F, Rustad CF, Riess A, Đukić D, Hsieh TC, Jobani I, Prescott T, Bevot A, Erger F, Houge G, Redfors M, Altmueller J, Stokowy T, et al. The Discovery of a LEMD2-Associated Nuclear Envelopathy with Early Progeroid Appearance Suggests Advanced Applications for AI-Driven Facial Phenotyping. Am J Hum Genet. 2019; 104:749–57. 10.1016/j.ajhg.2019.02.021 30905398 PMC6451726

[47] Scheibye-Knudsen M, Scheibye-Alsing K. 2012. “Welcome to the tutorial”. mitodb.com. 2022. http://www.mitodb.com/tutorial.html

[48] Das Angel. “Hierarchical Clustering in Python using Dendrogram and Cophenetic Correlation”. Towards Data Science. 2022. https://towardsdatascience.com/hierarchical-clustering-in-python-using-dendrogram-and-cophenetic-correlation-8d41a08f7eab

[49] Bonsai. “Software”. 2023. http://bonsai.hgc.jp/~mdehoon/software/cluster/software.htm

[50] Jolliffe IT, Cadima J. Principal component analysis: a review and recent developments. Philos Trans A Math Phys Eng Sci. 2016; 374:20150202. 10.1098/rsta.2015.0202 26953178 PMC4792409

[51] Groth D, Hartmann S, Klie S, Selbig J. Principal components analysis. Methods Mol Biol. 2013; 930:527–47. 10.1007/978-1-62703-059-5_22 23086856

[52] Insilico Medicine. “Panda Omics”. 2023. https://insilico.com/pandaomics

[53] Ozerov IV, Lezhnina KV, Izumchenko E, Artemov AV, Medintsev S, Vanhaelen Q, Aliper A, Vijg J, Osipov AN, Labat I, West MD, Buzdin A, Cantor CR, et al. *In silico* Pathway Activation Network Decomposition Analysis (iPANDA) as a method for biomarker development. Nat Commun. 2016; 7:13427. 10.1038/ncomms13427 27848968 PMC5116087

[54] Jassal B, Matthews L, Viteri G, Gong C, Lorente P, Fabregat A, Sidiropoulos K, Cook J, Gillespie M, Haw R, Loney F, May B, Milacic M, et al. The reactome pathway knowledgebase. Nucleic Acids Res. 2020; 48:D498–503. 10.1093/nar/gkz1031 31691815 PMC7145712

