# Supplementary material for: Defining the progeria phenome
Source: Aging (Albany NY). 2024 Feb 9;16(3):2026–46. doi: 10.18632/aging.205537 (PMC10911340; doi:10.18632/aging.205537)
Supplement: Supplementary Figures [file aging-16-205537-s001.pdf]

SUPPLEMENTARY FIGURES

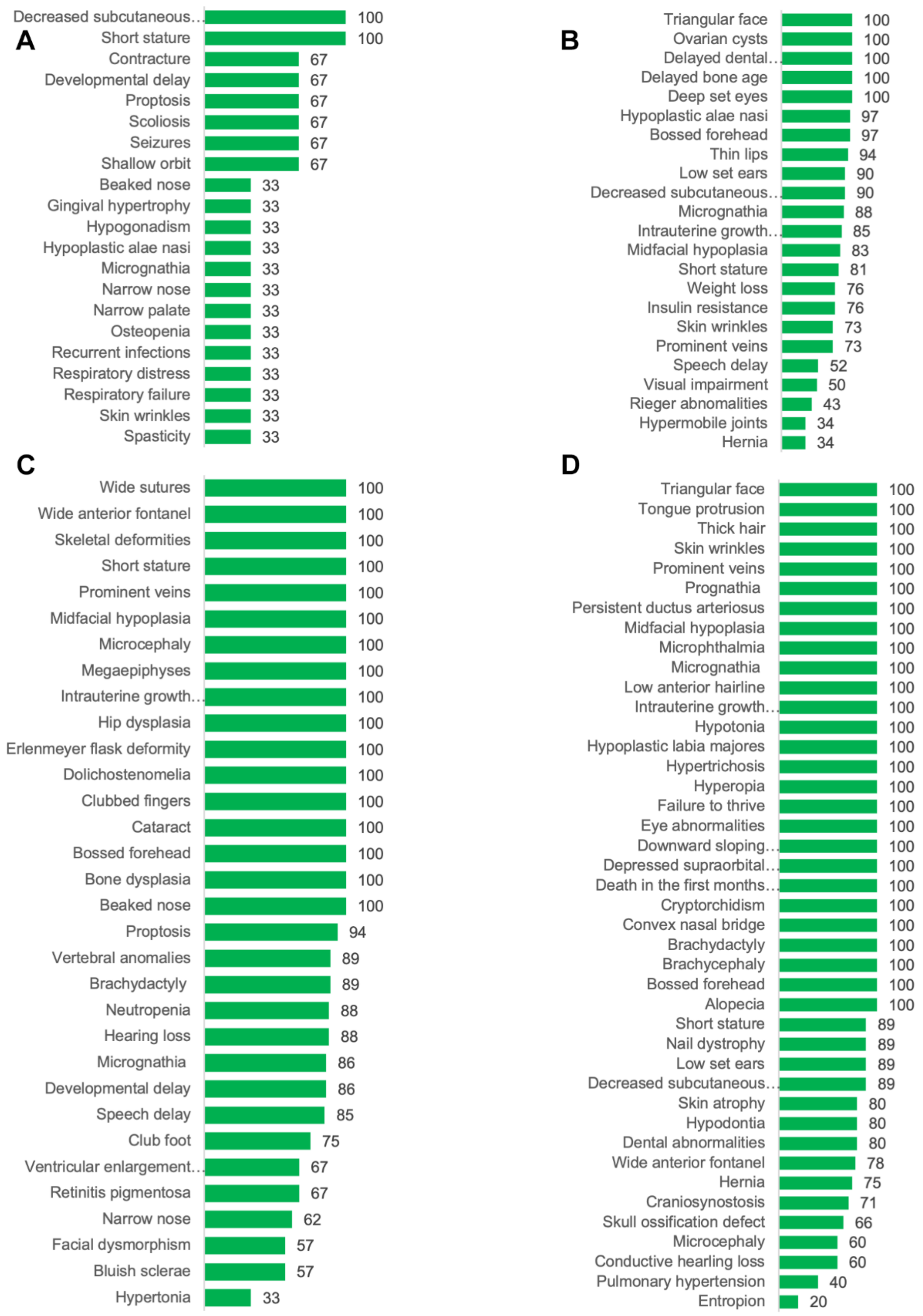

Supplementary Figure 1. Identification and visualization of phenotypes in identified progeroid syndromes. Bar charts showing the prevalence (%) of phenotypes for (**A**) Keppen-Lubinsky syndrome (KPLB), (**B**) SHORT syndrome, (**C**) Saul-Wilson syndrome and (**D**) Fontaine syndrome.

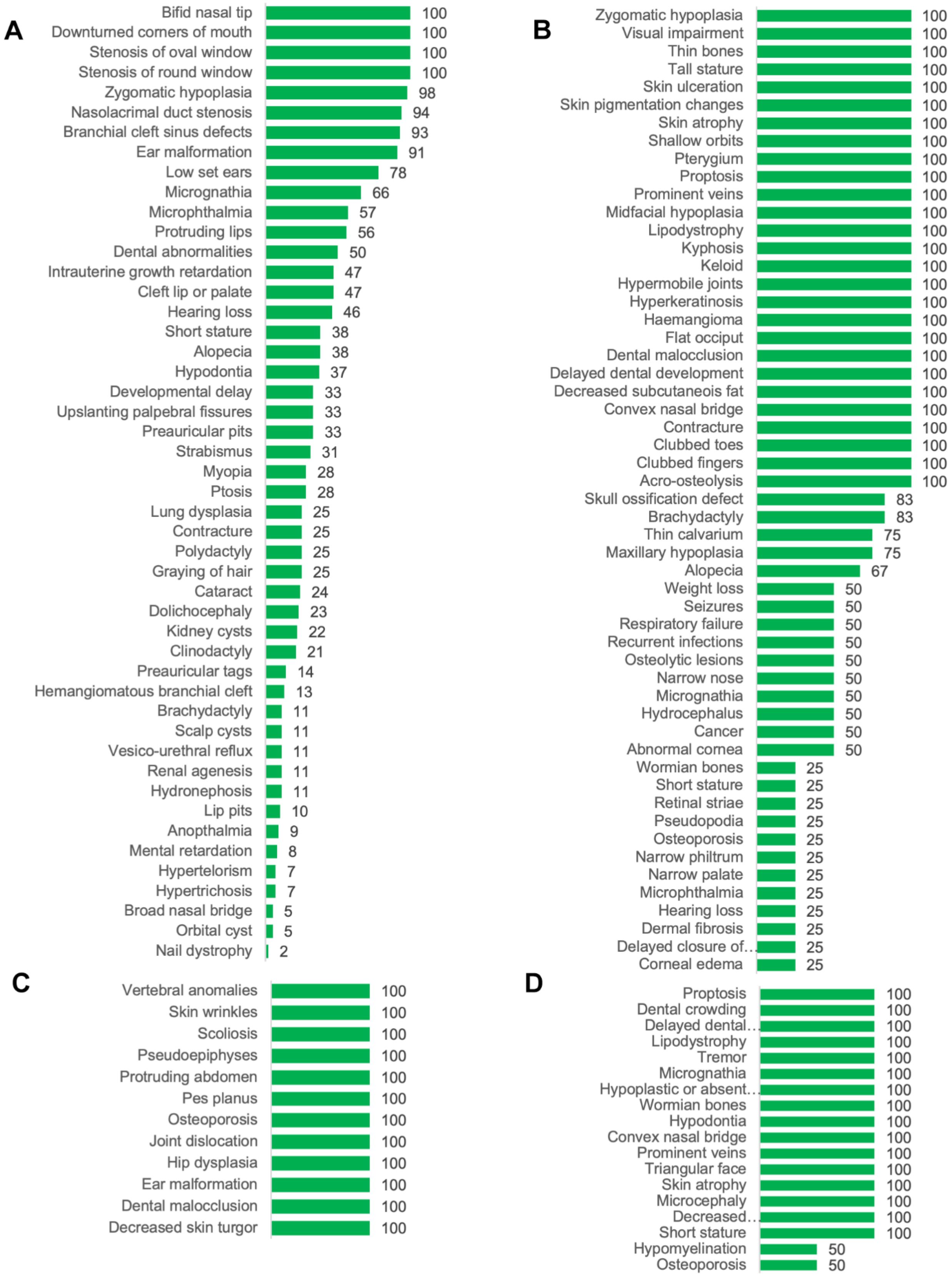

Supplementary Figure 2. Identification and visualization of phenotypes in identified progeroid syndromes. Bar charts showing the prevalence (%) of phenotypes for (**A**) Braciooculofacial syndrome (BOFS), (**B**) Penttinen syndrome, (**C**) Geroderma osteodysplasticum (GO), (**D**) Marbach-Rustad progeroid syndrome (MARUPS).

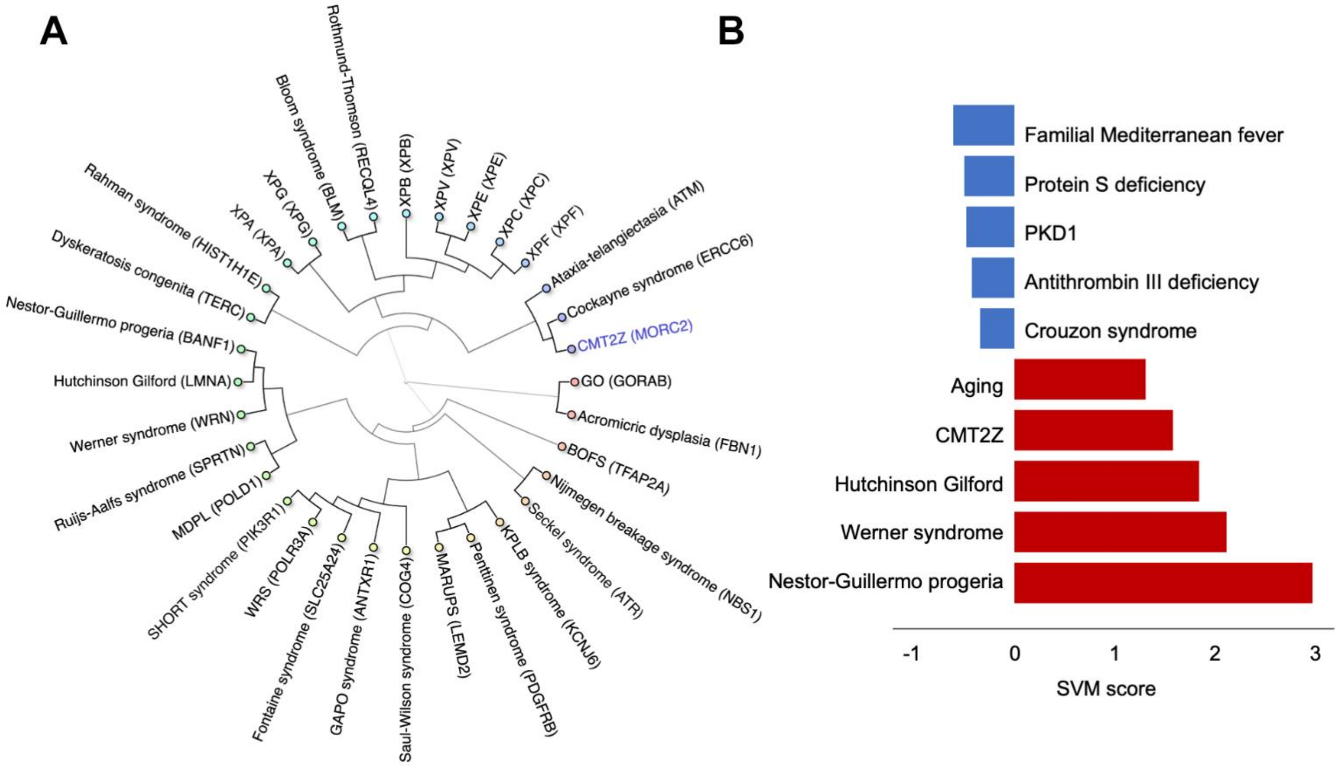

Supplementary Figure 3. Charcot-Marie-Tooth type 2Z (CMT2Z) may be a premature aging disorder. (**A**) Hierarchical clustering of premature aging diseases and CMT2Z. (**B**) Progeria SVM score of some premature aging diseases and CMT2Z.
